# Ischemic Conditioning to Reduce Fatigue in Isometric Skeletal Muscle Contraction

**DOI:** 10.3390/biology12030460

**Published:** 2023-03-16

**Authors:** Ruben Allois, Pasquale Pagliaro, Silvestro Roatta

**Affiliations:** 1 Department of Neuroscience, University of Turin, 10125 Torino, Italysilvestro.roatta@unito.it (S.R.); 2 Department of Clinical and Biological Sciences, University of Turin, 10043 Orbassano, Italy

**Keywords:** fatigue, tissue oxygenation, ischemic pre-conditioning, isometric contraction, endurance

## Abstract

**Simple Summary:**

Ischemic preconditioning (IPC) is a protective maneuver that alternates short periods of occlusion and reperfusion of tissue blood flow. Nineteen subjects were enrolled in one of the two groups, IPC (3 × 5/5 min right arm ischemia/reperfusion; cuff inflations 250 mmHg) and SHAM (3 × 5/5 min pseudo ischemia/reperfusion; 20 mmHg). The subjects performed a fatiguing contraction protocol before and 30 min after the IPC treatment. Results suggest that IPC may delay fatigue onset by reducing muscle oxygen consumption. The decrease in tissue oxygenation and the increase in deoxygenated hemoglobin were significantly reduced post- vs. pre-IPC (*p* < 0.05), but not post- vs. pre-SHAM. IPC delays the onset of fatigue, probably due to improved metabolic efficiency of muscles.

**Abstract:**

Ischemic preconditioning (IPC) is a non-invasive protective maneuver that alternates short periods of occlusion and reperfusion of tissue blood flow. Given the heterogeneity in the magnitude and frequency of IPC-induced improvements in physical performance, here we aimed to investigate, in a well-controlled experimental set-up, the local effects of IPC in exposed muscles in terms of tissue oxygenation and muscle fatigue. Nineteen subjects were enrolled in one of the two groups, IPC (3 × 5/5 min right arm ischemia/reperfusion; cuff inflations 250 mmHg) and SHAM (3 × 5/5 min pseudo ischemia/reperfusion; 20 mmHg). The subjects performed a fatiguing contraction protocol before and 30 min after the IPC treatment, consisting of unilateral intermittent isometric elbow flexions (3 s ON/OFF, 80% of maximal voluntary contraction) until exhaustion. While muscle strength did not differ between groups, post- vs. pre-treatment endurance was significantly reduced in the SHAM group (4.1 ± 1.9 vs. 6.4 ± 3.1 repetitions until exhaustion, *p* < 0.05) but maintained in IPC (7.3 ± 2.0 vs. 7.1 ± 4.3, n.s.). The decrease in tissue oxygenation and the increase in deoxygenated hemoglobin were significantly reduced post- vs. pre-IPC (*p* < 0.05), but not post- vs. pre-SHAM. The results suggest that IPC delays the onset of fatigue likely through improved metabolic efficiency of muscles.

## 1. Introduction

Ischemic preconditioning (IPC; brief cycles of artery occlusion/reperfusion) is a phenomenon conferring some degree of protection towards future episodes of ischemia followed by reperfusion [1]. IPC is operative in all species tested so far, including humans, and reduces infarct size in the studied organ through the release of trigger molecules and activation of intracellular signal signaling [2,3,4] to protect local or systemic organs against subsequent ischemia [5]. This phenomenon was first discovered and investigated in the cardiac muscle, and it was later shown to exist in other organs as well. Moreover, a similar protection could also be evoked by ischemia in remote tissues/organs (remote ischemic preconditioning, RIPC), leading to the concept that IPC/RIPC may be beneficial to every tissue of the body [6]. With a few exceptions, neither mechanical nor pharmacological protective interventions have been successfully translated from preclinical animal experiments to better clinical outcomes in recent phase III trials in patients [7,8].

Besides clinical applications, IPC has been proposed as a technique to improve muscle activity [9] and exercise performance [10] in healthy [11] and diseased populations [12,13]. Most often implemented as a sequence of 3–4 ischemic stimuli lasting several minutes and separated by reperfusion intervals of a few minutes, IPC was shown to improve maximal oxygen consumption by 3% and power output by 1.6% in an incremental bicycle exercise test [14]. In other studies, increments in performance were reported to range between 1% and 8% in various sports disciplines [15], including cycling [16], running [17] and static/dynamic apnea [18]; although, a recent meta-analysis pointed out that in many studies a proper control group was missing and that reported improvements in performance were generally small, with little practical significance [19]. However, underlying mechanisms and mediators are still largely unknown [20]. In particular, improvement in overall sports performance could be mediated both by local effects in the affected muscles as well as systemic effects [21]. While it is important to conduct field studies on sports activity [19] to quantify the overall benefit of the treatment, carefully controlled experimental conditions may be preferred to investigate local changes in working muscles. Several investigations have attempted to disentangle the interdependence among muscle force production, metabolism, tissue perfusion and oxygenation by adding near infrared spectroscopy (NIRS) measurement for monitoring tissue oxygenation or local O_2_ consumption, but conflicting observations have been reported. In some cases, increased performance after IPC was associated with or attributed to increased O_2_ consumption [19,22,23] or lactate production [24], while other studies have evidenced the opposite effect of IPC [17,25]. The reason for controversial results could be due to differences in experimental conditions and protocols but leaves a basic question unanswered: does IPC potentiate the muscle capacity to deliver force and power or, rather, increase the resistance to fatigue by increasing the efficiency of the contractile machinery and lowering O_2_ consumption?

The present study aims to examine the issue with a novel approach in a laboratory setting, with a focus on performing and controlling the exercise task (an intermittent isometric contraction of the biceps brachii to exhaustion) in terms of developed strength and muscle activation, continuously monitoring local NIRS variables.

## 2. Materials and Methods

### 2.1. Subjects and Study Design

Nineteen subjects volunteered, all right-handed, to participate in this study and were divided into two groups to follow two different protocols: IPC or placebo (SHAM). The IPC group comprised ten healthy subjects, eight males and two females (age: 29 ± 6 years, weight: 73 ± 6 kg, height: 173 ± 10 cm; BMI: 23.42 ± 1.5 kg/m^2^) and the SHAM group included nine healthy subjects, seven male and two female (age: 26 ± 4 years, weight: 72 ± 5 kg, height: 172 ± 7 cm; BMI: 24.32 ± 0.3 kg/m^2^). All subjects were non-smokers, were not taking medications or supplements, were not being treated for orthopedic injuries and did not present with a history of chronic disease. Five subjects (subjects 3, 6, 7 in the IPC group and subjects 1 and 9 in the SHAM group) were routinely training in the gym. The study was approved by the institutional ethics committee of the University of Torino. Each subject gave written informed consent.

### 2.2. Experimental Set-Up

**Figure 1:**
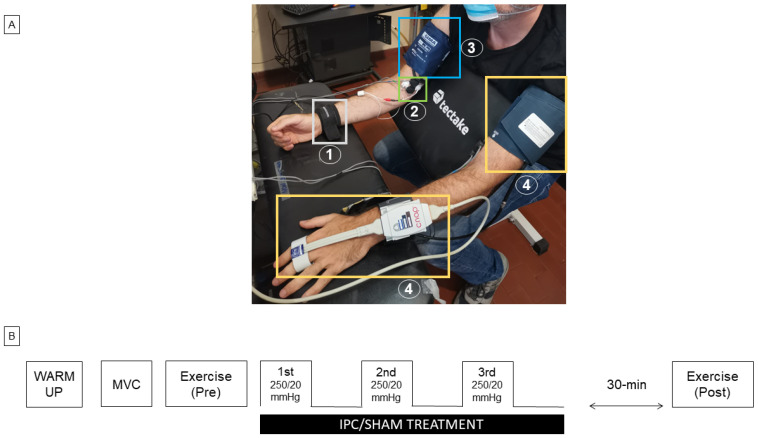
Experimental Setup (**A**). During the measurements, subjects were sitting on an adapted armchair, specific for the fitness training. The subject rested his forearms on a horizontal support. A load cell (not visible) was connected to the right wrist by a wristband (1). Near infrared spectroscopy probe and EMG electrodes (2) were located on the distal end of the bicep’s brachii muscle, and the pneumatic cuff for ischemic pre-conditioning was located proximally on the right arm (3). Non-invasive blood pressure monitoring was applied to the left arm at the fingers, with a pneumatic cuff around the arm for calibration (4). Schematic representation of the experimental protocol (**B**). MVC = maximal voluntary contraction; IPC = ischemic preconditioning: 3 × (5 min ON, 5 min OFF, cuff pressure: 250 mmHg); SHAM = sham intervention: 3 × (5 min ON, 5 min OFF, cuff pressure: 20 mmHg). The exercise consisted of unilateral intermittent isometric elbow flexions (3-s ON, 3-s OFF) at 80% MVC until exhaustion.

A weightlifting bench was modified to implement isometric contractions of the biceps muscle. As can be observed in Figure 1A, the subject could sit, leaning forward a bit, with the trunk and arms resting against the supports as this arrangement effectively kept the upper arm and the shoulder still during elbow flexion. In addition, horizontal support for the forearm was added to the bench; the height of the two supports and the seat were individually adjusted to achieve 120-deg flexion of the elbows. The right wrist was enwrapped by a wrist band, rigidly connected through a tightened strap to a load cell (50 kg) anchored to the ground. The calibrated force signal could be displayed on a screen and used as visual feedback for the subject during the exercise. The IPC (or SHAM) was performed on the right arm using a pneumatic cuff (17.5 × 36 cm, Gima, Italy) to occlude the brachial artery. The cuff was rapidly inflated to 250 mmHg (or to 20 mmHg for SHAM) and rapidly deflated to 0 mmHg after 5 min, using a computerized system, operating on a proportional valve (ITV1010, RTI s.r.l., Torino, Italy) [26,27]. Arterial blood pressure (ABP) was continuously and non-invasively monitored by finger photo-plethysmography (CNAP system, CNSystems Medizintechnik, Graz, Austria), along with cardiac output (CO) and heart rate (HR). The device was periodically recalibrated by cuff measurement at the left arm. Near-infrared spectroscopy (NIRS) (NIRO-200X, Hamamatsu Photonics K.K, Shizuoka, Japan) was adopted for the non-invasive monitoring of muscle oxygenation. The NIRS probe (inter-optode distance of 3 cm) was bilaterally located distally to pneumatic cuffs, and longitudinally oriented, to limit the curvature of the probe over the distal end of the biceps muscle. The device measures changes in time in oxygenated (O_2_Hb) and deoxygenated (HHb) hemoglobin + myoglobin, expressed in µM*cm, according to the standard Beer–Lambert methodology, as well as the tissue oxygenation index (TOI) and tissue hemoglobin index (THI), representing the oxygen-saturated hemoglobin + myoglobin in percentage, and the total hemoglobin + myoglobin concentration in arbitrary units, respectively, computed according to the spatially resolved methodology. This latter technique more specifically focuses on the measurement in the deep muscle tissue and is less affected by hemodynamic changes in superficial cutaneous tissue layers [28,29]. Bipolar surface electromyographic (EMG) recording was measured from the right brachial biceps (QUATTRO, OT Bioelettronica Srl, Torino, Italy). After cleansing the skin with an abrasive prepping-gel the electrodes (NUPREP, D.O Weaver and Co, Aurora, CO, USA) were applied to the biceps muscle. To match the EMG with the NIRS sample volume, the EMG electrodes with an inter-electrode distance of approximately 3 cm were placed transversally to the NIRS probe [30] and, thus, also to the muscle. All signals were digitally sampled at frequencies of 10 Hz (ABP, CO, HR, and NIRS variables), 100 Hz (Force and cuff pressure), and 2 kHz (EMG) by a single acquisition board (1401 micro, CED, Cambridge, UK), then stored on a PC for offline analysis (Spike2, CED, Cambridge, UK). Data acquisition was continuously performed throughout the experimental session.

### 2.3. Experimental Protocol and Exhaustion Detection

**Figure 2:**
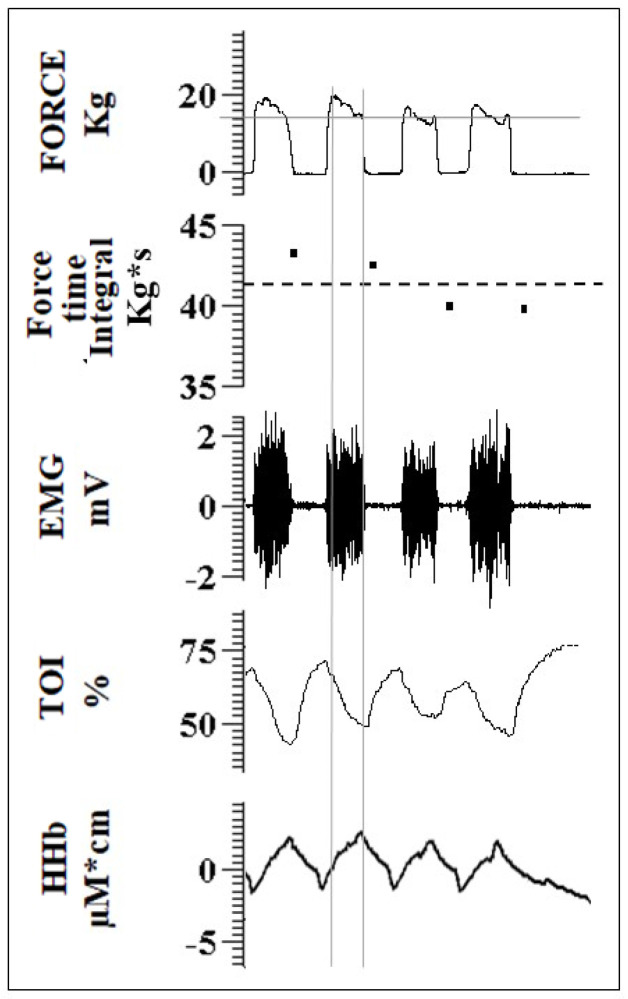
Signals during exercise. From top to bottom: Exerted force, the dotted line corresponding to 80% MVC—this signal was provided as visual feedback to the subject; force–time integral calculated in real-time over each single contraction, the dashed line corresponding to 90% of the expected force–time integral calculated over one contraction (=80% MVC * 3 kg*s) (see text)—this signal, checked by the experimenter, drops below the threshold if the force is too low and/or the duration of the contraction is too short; electromyographic (EMG) signal from the bicep muscle; tissue oxygenation in bicep muscle (TOI); changes in deoxyhemoglobin concentration (HHb). Vertical gray continuous lines indicate the interval from 0.5–3-s from the beginning of the contraction, used to calculate EMG and NIRS variables during exercise.

A scheme of the experimental protocol is given in Figure 1B. The subject was familiarized with the set-up, the visual force feedback, and with performing rhythmic isometric elbow flexion according to an acoustic pace (3-s ON; 3-s OFF). To this aim, they were invited to follow a warm-up: 3 sets of 10 repetitions at low force intensity (1st set: 2 kg; 2nd set: 3 kg; 3rd set: 5 kg). They were then asked to perform a Maximum Voluntary Contraction (MVC), which was repeated another two times separated by a 2-min rest. The maximum force level observed, after smoothing the tracing with a 1-s moving average, was taken as the MVC. The fatiguing contraction protocol consisted of a series of unilateral (right) intermittent isometric elbow flexions (3 s ON and 3 s OFF, according to the acoustic cue) at 80% MVC, continued until exhaustion. This contraction level was chosen to reach exhaustion within a reasonable amount of time, while a duration of 3 s appeared to be long enough to reach a stable and controllable force level and stable hemodynamic signals within individual contractions. The force signal was carefully monitored during the exercise: the extent of every single contraction was assessed in terms of magnitude and duration to provide a real detection of fatigue as follows. Visual feedback of the exerted force (low-pass filtered with a 1-s moving average) with an indication of the level (80% MVC) to be reached was provided to the subject. However, to also check for the duration of each contraction (ideally = 3 s), the force–time integral (ideally = 3* 80% MVC kg*s) was simultaneously computed over the whole duration of each contraction bout and visually compared to a threshold level equal to 90% of the expected value, i.e., 0.9* 3* 80% MVC, and expressed in kg*s (see second tracing from top in Figure 2). Failure to maintain this value above the threshold for two consecutive bouts determined the end of the task (exhaustion). This latter condition was monitored by the experimenter and verbally communicated to the subject. To induce IPC (or SHAM), 5 min of inflation and 5 min of deflation of the cuff were repeated three times unilaterally on the right arm. Thus, the IPC (or SHAM) procedure lasted for 25 min. After a 30-min resting time, following the last IPC (or SHAM) cuff inflation, the fatiguing contraction protocol was repeated with identical settings until exhaustion.

### 2.4. Data Analysis

Changes exhibited by hemodynamic and EMG variables during the exercise were measured at each individual contraction in terms of TOI, HHb, mean frequency (MNF) of the power spectrum and power of the EMG signal. The MNF was calculated as the average frequency of the power spectrum (fast Fourier transform size: 1024 pts, corresponding to epochs of 512 ms, calculated every 0.1 s by overlapping subsequent epochs by approximately 80%) over the range from 30–500 Hz, while the signal power was calculated as the integral of the power spectrum over the same frequency range. The first 0.5 s of each contraction were excluded from the analysis due to the possible occurrence of movement artifacts. Average values were, thus, calculated within the interval from 0.5–3 s from the beginning of each contraction as illustrated by the vertical lines in Figure 2.

For each subject, exercise-induced changes were calculated concerning the beginning of exercise (first contraction) and both at exhaustion (last valid contraction) and ISOtime, defined as the minimum exhaustion time observed in the pre- and post-treatment conditions. The assessment at ISOtime is meant to provide a comparison of effects after the same exercise duration since exhaustion could occur at different times from the beginning of the exercise in the pre- and post-treatment conditions. Analysis of the responses to the three-cuff inflation/deflation inducing ischemia/reperfusion was performed as follows. The speed of the TOI decrease (TOI slope) at the beginning of each stimulus was computed within the interval from 0–30 s from the beginning of the ischemic occlusion and expressed as absolute TOI change per second. The reactive hyperemia developing at reperfusion was assessed in terms of mean tissue oxygenation achieved over the first 2 min after the release of the occlusion. All signal processing was performed with the acquisition and analysis software Spike2 and measured values were collected in Excel sheets. Possible changes in blood volume introduced by the IPC/SHAM treatments were assessed by measuring THI over 10-s intervals taken immediately before and after the exercise.

### 2.5. Statical Analysis

Statistical analysis was performed using SPSS 27.0 (SPSS Inc., Chicago, IL, USA). The data sets were analyzed and the results are presented as the mean/standard deviation. The *p*-values less than 0.05 were considered significant. A one-way repeated measures ANOVA was conducted to determine whether there were statistically significant differences in ischemic compression in the Slope of Tissue Oxygenation Index (Slope TOI) in the first 30 s and hyperemia post-IPC. Given the small sample size, non-parametric tests were used. Comparisons within the IPC and SHAM groups were assessed with the Wilcoxon test to test differences between pre-and and post-treatment or taking place during exercise, e.g., last versus first contraction. Comparisons between IPC and SHAM groups were assessed with the Mann–Whitney test to test differences in maximum voluntary contraction, anthropometric variables, resting values of local and systemic variables, and treatment effects on exercise-related changes in hemodynamic variables.

## 3. Results

The IPC and SHAM groups did not differ in age, weight, height, BMI, or for resting values of HR (IPC: 77.6 ± 10.5 vs. SHAM: 71.9 ±10.8 bpm, n.s.), CO (5.4 ± 1.2 vs. 6.6 ± 1.7 L/min, n.s.), and TOI (6.60 ± 1.67 vs. 67.31 ± 5.64%, n.s.). Muscle strength, in terms of MVC, did not differ between groups (IPC: 19.9 ± 5.4 kg; SHAM: 21.6 ± 8.8 kg). In terms of endurance, quantified as the number of contractions before exhaustion, the results showed no difference pre- vs. post-treatment in the IPC group (post: 7.3 ± 2.7 vs. pre: 7.1 ± 4.3, n.s). On the contrary, in the SHAM group, the endurance decreased from 6.4 ± 3.1 in the pre-treatment test to 4.1 ± 1.9 reps (*p* < 0.05) in the post-treatment test.

### 3.1. Hemodynamic Changes during Exercise

**Figure 3:**
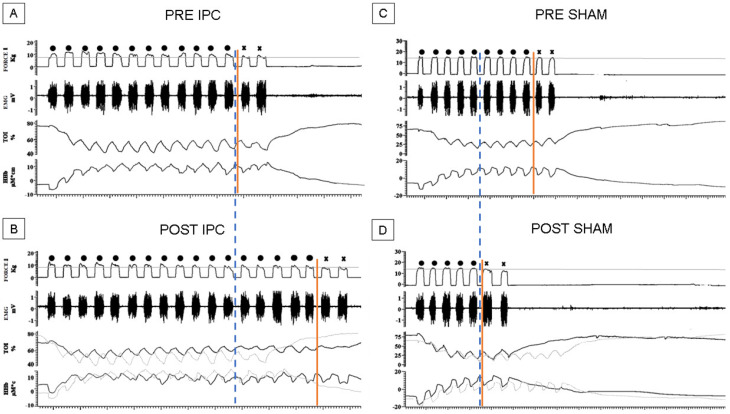
Exercise task. Original recordings from two representative subjects of the exercise task performed pre- (**A**,**C**) and post- (**B**,**D**) the IPC (**A**,**B**) or SHAM (**C**,**D**) treatment. Vertical lines indicate exhaustion time (continuous red line) and ISOtime (dashed blue line), i.e., the shortest exhaustion time in the pre- and post-treatment tasks. The dots over the force signal indicate valid contractions while the “X” indicates below-threshold contractions (after exhaustion).

We then investigated hemodynamic and EMG changes during the exercise. Figure 3 shows representative recordings from two subjects belonging to the IPC (Figure 3A,B) and the SHAM group (Figure 3C,D) describing the exercise before (A, C) and after treatment (B, D). As it can be observed, in these subjects, the performance could change substantially pre-and post-treatment along with the time course of NIRS parameters.

**Figure 4:**
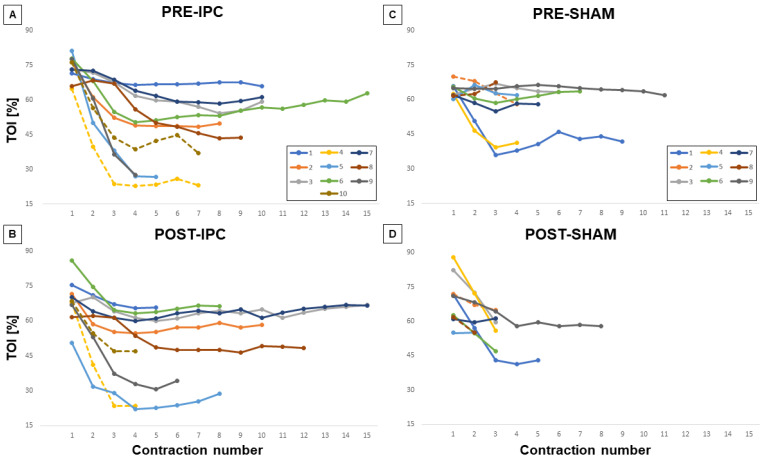
Individual changes in TOI during exercise. Each line corresponds to a different subject and each dot represents an individual contraction during the exercise task for the two groups IPC (**A**,**B**) and SHAM (**C**,**D**) and the pre- (**A**,**C**) and post-treatment conditions (**B**,**D**). Dashed lines refer to the individual curves of female subjects.

**Figure 5:**
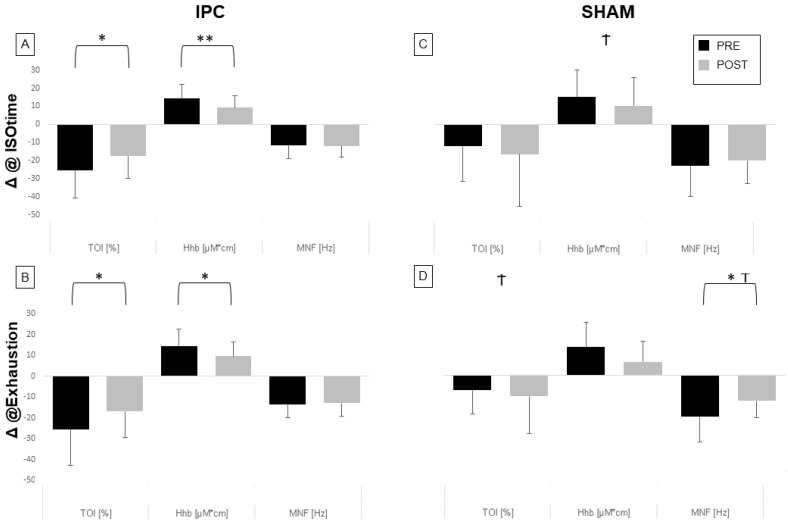
Average changes in TOI, HHb, and MNF during exercise. The changes are calculated at ISOtime (**A**,**C**) and at exhaustion (**C**,**D**) with respect to the first contraction for the two groups IPC (**A**,**B**), and SHAM (**C**,**D**) and pre- (black) and post- (gray) treatment conditions. * = pre vs. post, *p* < 0.05; ** = pre vs. post, *p* < 0.01. Average effects of treatment (post—pre) were compared between IPC and SHAM: † = *p* < 0.05.

Values for TOI (Figure 4), HHb, and MNF were calculated for each single exercise bout so that time courses could be described for each individual subject, respectively, while changes exhibited by these variables at ISOtime or at exhaustion (compared to first contraction bout) in the different conditions are compared in Figure 5.

In general, the TOI exhibited an initial decrease, which subsequently stabilized or slightly recovered before the end of the exercise. We observed that the decrease in TOI was significantly reduced post-IPC vs pre-IPC, both at ISOtime (17.6 ± 12.2% vs. 25.4 ± 15.4%, *p* < 0.05, 5A) and at exhaustion (16.9 ± 12.5% vs. 25.6 ± 17.4%, *p* < 0.05, Figure 5B). Conversely, no effect of treatment was observed in the SHAM group (Figure 5C,D), with a significant difference between the IPC and SHAM groups at exhaustion (*p* < 0.05). Similar effects were observed on HHb, which generally increased during exercise. The HHb increase was smaller post-IPC compared to pre-IPC, both at ISO-time (*p* < 0.01, Figure 5A) and at exhaustion (*p* < 0.05, Figure 5B), while no significant change was observed in the SHAM group (Figure 5C,D), with a significant between-group difference at ISOtime.

### 3.2. EMG Changes during Exercise

As for the EMG signal, MNF generally showed a decreasing trend with exercise. The magnitude of the MNF decrease was not affected by IPC, while it was significantly reduced during SHAM at exhaustion (*p* < 0.05, Figure 5D), with a significant difference between groups (*p* < 0.05). However, this effect could be attributed to a considerably reduced duration of the task in the post- vs. pre-SHAM condition.

Finally, relative changes in the intensity of muscle contraction were investigated by assessing the average power of the EMG signal calculated from the beginning of the task to ISO-time. No significant changes were produced by IPC or SHAM (12 ± 5.7%, post- vs. pre-IPC, n.s.; 38 ± 12.3%, post- vs. pre-SHAM).

### 3.3. Hemodynamic Changes during Treatment

**Figure 6:**
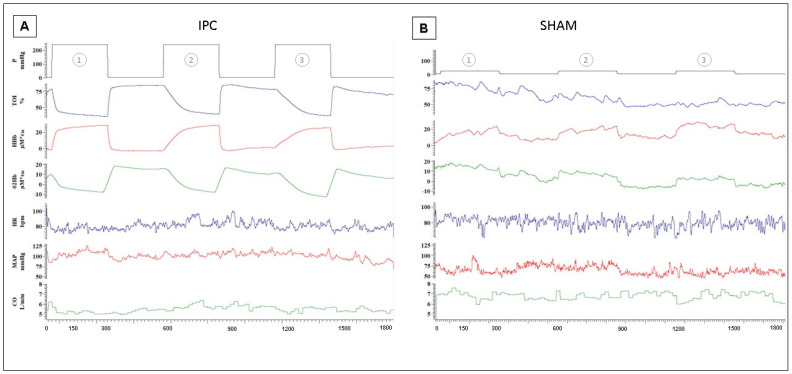
Hemodynamic responses to 1st (1), 2nd (2), and 3rd (3) compressive stimuli in IPC (**A**) and SHAM (**B**) in two representative subjects. From top to bottom: Cuff Pressure (P); tissue oxygenation index (TOI); changes in deoxy-hemoglobin (HHb = deoxHb + deoxyMb), oxy-hemoglobin concentration (O_2_Hb = oxyHb + oxyMb), heart rate (HR); arterial blood pressure (MAP) and cardiac output (CO).

Original recordings from a representative subject of the local hemodynamic response to IPC treatment are shown in Figure 6A, as compared to the response to SHAM (Figure 6B).

The expected effects of ischemia are visible in terms of a decrease in TOI and O_2_Hb and an increase in HHB. Note also that the time course of changes is different in the three subsequent ischemic stimuli. The progressively slower time course of the response to IPC was quantified by the initial slope (estimated at from 10–30 s, from the start of ischemia) of the TOI curve, which was significantly higher during IPC1 (−1.07 ± 0.66%/s) compared to IPC2 (0.45 ± 0.29%/s, *p* < 0.01) and IPC3 (0.33 ± 0.22%/s, *p* < 0.01). We also observed that the tissue oxygenation during reactive hyperemia (at reperfusion) significantly decreased after IPC3 (74.17 ± 8.49%, *p* < 0.01) compared to IPC1 (81.58 ± 6.16%). As for the SHAM treatment, no meaningful changes were observed in TOI and only small upward deflections were exhibited by O_2_Hb and HHb, revealing the small increase in blood volume caused by the slight venous occlusion. Finally, to investigate whether the treatments introduced possible alterations in the vascular setting we compared THI post- vs. pre-treatment and found no significant changes with IPC (+1.2 ± 11.8%) or SHAM (−4.7 ± 7.7%).

## 4. Discussion

In the present study, we demonstrated that ischemic preconditioning reduces fatigue in isometric skeletal muscle contraction. We investigated whether ischemic preconditioning affects motor performance and whether the effects can be explained by local changes in hemodynamic and EMG variables in a controlled laboratory setting. The results showed that the time to exhaustion in two subsequent intermittent isometric elbow flexion tasks was unchanged if IPC was performed in between the tasks, whereas it was significantly reduced in the case of SHAM treatment. We observed that the decrease in tissue oxygenation and the increase in the concentration of deoxygenated hemoglobin, which took place during the task, were significantly reduced after IPC and not after SHAM (both at ISOtime and at exhaustion), with significant group differences, while no significant group difference was observed in EMG variables at ISOtime.

### 4.1. Effects of IPC on Exercise

Worsening of endurance in the SHAM group indicates that the fatigue accumulated with the first exercise bout was not completely recovered after 1 h (30 min of treatment + 30 min of rest). Conversely, the performance was unchanged in the IPC group, suggesting that the effect of the IPC treatment effectively counteracted the residual impairment of muscle function. Improvement in endurance has been observed in other studies [24,31], although not all of them [32,33]. In principle, the effects of IPC could be exerted both at the central/systemic level or locally, in the engaged muscles. The analysis of recorded variables sheds some light on the underlying mechanisms.

Central effects. It has been suggested that the SHAM group would not adequately protect from possible placebo effects in investigations about the possible ergogenic effect of IPC [19]. In the present study, to further limit the possible placebo effect, we did not mention to the subjects that the IPC treatment could be beneficial. In addition, the analysis of EMG variables did not evidence any difference in the extent of muscle activation during the exercise post- vs. pre-IPC (or SHAM), suggesting no change in the descending motor drive.

Local effects. Interestingly, NIRS monitoring revealed that tissue oxygenation during the exercise decreased less in post- vs. pre-IPC and this improvement was observed both after a comparable number of contractions (ISOtime) and at exhaustion. Such improvement in tissue oxygenation could result from increased perfusion and/or decreased oxygen consumption. Although blood flow to the bicep muscle was not measured, increased perfusion, mediated by local dilatory mechanisms is generally associated with local increases in blood volume. These can be effectively detected by NIRS as previously observed in different conditions, such as stress-induced dilatation in the masseter muscle [34], contraction-induced hyperemia of bicep brachii [28] and forearm muscles [29] as well as cutaneous dilatory hyperemia induced by local warming [28,29]. On this basis, unchanged pre-exercise THI levels following either IPC or SHAM treatments suggest that basal muscle perfusion levels were not affected and, consequently, that the explanation for the increased tissue oxygenation during the exercise after IPC is to be attributed to decreased oxygen consumption by muscle fibers. This is in accordance with what was suggested by Orbegozo Cortés D [35]. Our interpretation is further supported by the observation that the increase in HHb during exercise was reduced after IPC and not after SHAM. In fact, HHb is considered to be a reliable indicator of oxygen consumption (during ischemia) and of fractional O_2_ extraction in constant work-rate exercise [36] and to correlate to myoelectric fatigue [30], while being less affected by changes in cutaneous circulation, as compared to O_2_Hb. The concept that IPC might decrease oxygen consumption in muscles is only apparently in contrast with a number of studies reporting increased metabolism [23,37], increased lactic acid production [24], and increased muscle deoxygenation [22,38]. In fact, in some of these studies, the comparison post- vs. pre-IPC was not performed at the same muscle load and the increase in metabolism was observed in association with increased muscle activity, quantified in terms of force [38] or EMG [24]. In other studies, a faster increase in the HHb curve during exercise was reported post- vs. pre-IPC [22,23]; however, the observation was made after normalizing the HHb curves to the maximum value (achieved at the end of exercise) while original non-normalized curves evidence that such maximum value is actually smaller post- vs. pre- IPC, in agreement with the present data.

### 4.2. Response to the Subsequent Ischemic Stimuli of IPC

Additional evidence of IPC-induced reduction in metabolic rate in the muscle is provided by NIRS monitoring during the IPC treatment. In fact, the rate of oxygen consumption is reflected by the descending slope of TOI during ischemia [36]. Orbegozo-Cortes reported that the TOI descending slope decreased by approximately 15% during the second vascular occlusion test that lasted 3 min compared to the first, irrespective of the time lag (5–30 min) between the two [35]. This effect was confirmed in the present study.

A reduction in the metabolic rate of the resting muscle by IPC is also expected to cause smaller reactive hyperemia to the ischemic stimulus. This was, in fact, reported to occur: a decreased blood oxygenation level-dependent (BOLD) signal in the hyperemic response to a complex protocol including ischemia, active muscle contraction, and venous occlusion was observed after IPC, although differently interpreted [37]. The changes in TOI may reflect differences in perfusion, oxygen consumption, and demand. In the present study, we monitored the time course of TOI during both the ischemic and the reperfusion phases and considered the overshoot of TOI at reperfusion as an indicator of reactive hyperemia. In fact, TOI, as a spatially-resolved NIRS variable, was shown to be a reliable indicator of oxygenation of deep tissues [29,39] and to systematically increase in proportion to the magnitude of hyperemia in the muscle [40]. During the present IPC treatment, we observed a progressively decreased magnitude of the reactive hyperemia. In agreement with this latter observation, a progressively shorter-lasting hyperemic response to three subsequent ischemic stimuli was reported by Mota et al. in their Figure 2 [33], although not specifically discussed.

Although the effects observed in the present study were probably overestimated, due to the fact that IPC was started shortly after the exercise, the trends agree with the other studies in the literature and support an IPC-induced reduction in the metabolic rate of the muscle tissue, again supporting our view that IPC induces a reduction in oxygen demand and consumption, thus, improving the efficiency of muscle metabolism.

### 4.3. Experimental Set-Up Advantages and Limitations

A few features were implemented aimed at improving the accuracy of the investigation: (1) the modified weightlifting bench, providing support for both the forearm, upper arm, and trunk, minimized the involvement of muscles other than the elbow flexors; (2) the double control of force magnitude and duration for individual contractions of the isometric exercise excluded the possibility that increased endurance could be achieved by performing contractions shorter than required (as indicated by the acoustic cue); (3) both EMG electrodes and NIRS optodes continuously recorded the signals throughout the whole experimental session, thus, excluding the possible inaccuracy associated with repositioning, as required in multiple-session studies; (4) analysis of EMG and NIRS variables was performed at each individual contraction and, over time, intervals limited within the stable contraction phase, rather than averaging over large intervals that included contraction and relaxation phases in which movement artifacts and large changes in muscle blood flow may have disturbed the measurement. Adopting these features allowed for accurate comparison of measurements collected pre- and post-treatment.

The limitation of this approach is that two exhausting exercises were performed within the same session. Although more than 1 h separated the pre- from the post-treatment exercise, long-lasting fatigue may have affected the performance in the post-treatment exercise. However, this bias equally affected the two groups and did not prevent the appearance of group differences. In addition, here we explored a single force level (80% MVC) at which virtually all muscle fibers are recruited. Differential effects of IPC on different fiber types (glycolytic/oxidative) could affect the results observed at low or high contraction levels, associated with preferential recruitment of oxidative or both oxidative and glycolytic fiber types, respectively. A further limitation of the study is the small sample size, which does not allow to draw conclusions about the influence of training status and sex on the effects of IPC. By observing individual curves in Figure 4, we can notice that, as expected, physically active subjects tend to exhibit higher resistance to fatigue, and that the 2 females in the IPC group did not exhibit performance improvement after treatment. This latter observation is in line with the report by Paradis-Deschênes et al., which describes ergogenic effects of IPC in males but not in females during maximum voluntary knee extensions [38]. These possibilities and limitations could account for part of the variability observed in the literature and will need to be investigated in future studies.

## 5. Conclusions

In conclusion, this study, in which a controlled constant-force intermittent isometric contraction was investigated, suggests that IPC introduces functional changes in the exposed skeletal muscles. The results collected during both the preconditioning treatment and the exercise consistently point to an IPC-induced improvement in the efficiency of muscle metabolism, both at rest and during 80% MVC contractions. These effects likely explain the capacity to counteract the lasting effects of an exhausting exercise, exhibited by the IPC and not by the SHAM group.

## Data Availability

The data that support the findings of this study are available within the manuscript and from the author (S.R.) upon request.
